# Protease Supplementation Partially Alleviates the Negative Effects of Low-Protein Diets on Growth Performance in Weaned Piglets

**DOI:** 10.3390/vetsci13070616

**Published:** 2026-06-25

**Authors:** Wei Han Zhao, Si Yeong Choi, In Ho Kim

**Affiliations:** 1 Department of Animal Biotechnology, Dankook University, Cheonan 31187, Republic of Korea72231558@dankook.ac.kr (S.Y.C.); 2 Smart Animal Bio Institute, Dankook University, Cheonan 31187, Republic of Korea

**Keywords:** protease, weaned piglets, low-protein diet, growth performance, nutrient digestibility, fecal score

## Abstract

This study evaluated the effects of reducing dietary crude protein (CP) levels and protease supplementation on growth performance, nutrient digestibility, and fecal score in weaned piglets. A total of 200 crossbred weaned piglets (Duroc × [Landrace × Yorkshire]), with an initial body weight (BW) of 6.01 ± 1.14 kg, were used in a 31-day feeding trial. Piglets were assigned to four dietary treatments in a randomized complete block design with 10 replicates per treatment and five piglets per pen. The treatments were as follows: CON, basal diet; TRT1, low-protein diet with CP reduced by 1%; TRT2, low-protein diet with CP reduced by 2%; and TRT3, TRT2 supplemented with 0.1 g/kg protease. Piglets fed TRT2 had lower BW on days 7, 19, and 31 (*p* < 0.05) and lower average daily gain (ADG) during each growth phase and the overall period compared with CON (*p* < 0.05). Protease supplementation partially restored BW and ADG. However, average daily feed intake (ADFI), feed conversion ratio (FCR), apparent digestibility of dry matter (DM), nitrogen (N), and energy (E), and fecal score were not affected by dietary treatments (*p* > 0.05). In conclusion, reducing dietary CP by 2% impaired growth performance in weaned piglets, whereas protease supplementation partially alleviated this negative effect without significantly altering nutrient digestibility or fecal score.

## Simple Summary

Reducing protein levels in pig diets can lower feed costs and nitrogen waste, but excessive reduction may impair piglet growth after weaning. This study examined whether adding protease, an enzyme that helps digest protein, could improve growth in piglets fed reduced-protein diets. A total of 200 weaned piglets were fed different protein levels with or without protease for 31 days. Piglets fed the diet with a two percent protein reduction showed lower body weight and daily weight gain, whereas protease supplementation partly restored growth. Feed intake, feed efficiency, nutrient digestibility, and fecal condition were not affected. These results suggest that protease may help support piglet growth when dietary protein is moderately reduced, contributing to more efficient pig production.

## 1. Introduction

In modern intensive swine production systems, the level of dietary protein not only directly affects animal growth performance but is also closely associated with feed cost and environmental N emissions [1]. Conventional diets for piglets often contain relatively high levels of CP to meet the amino acid requirements during the rapid growth stage [2]. However, excessive dietary protein, particularly when exceeding the digestive capacity of piglets, may result in a greater proportion of undigested N reaching the hindgut, where it undergoes microbial fermentation and produces ammonia, amines, and other potentially harmful metabolites [3]. These compounds can increase environmental N pollution and may negatively affect intestinal health. Therefore, reducing dietary protein levels while maintaining a balanced supply of essential amino acids to improve N utilization efficiency has become an important focus in swine nutrition research [4].

Previous studies have shown that, when crystalline amino acids are adequately supplemented, moderate reductions in dietary protein can decrease N excretion and lower feed costs [5]. However, excessive reductions in dietary protein may limit amino acid availability and consequently impair growth performance in piglets [6]. This issue is particularly important during the post-weaning period, when the digestive system of piglets is still immature, and their capacity for protein digestion and amino acid absorption may be limited [7]. Under these conditions, reducing dietary CP may increase the risk of inadequate amino acid supply, especially for growth-related amino acids such as lysine, methionine, leucine, cystine, glutamic acid, alanine, and phenylalanine [8]. In this context, the use of exogenous enzymes in animal nutrition has received increasing attention [9]. Among them, proteases have been widely studied for their ability to enhance protein hydrolysis and improve amino acid digestibility [10]. Protease supplementation can hydrolyze dietary proteins and certain anti-nutritional factors, thereby increasing the release of absorbable amino acids in the small intestine [11]. Previous studies have reported that protease supplementation can improve the apparent ileal digestibility and standardized ileal digestibility of CP and specific amino acids, including lysine, methionine, leucine, cystine, glutamic acid, alanine, and phenylalanine [12]. In addition, proteases may reduce the flow of undigested protein to the hindgut, thereby potentially decreasing microbial fermentation, which in turn decreases the formation of harmful fermentation metabolites and may contribute to improved growth performance and intestinal health in animals [13].

Previous studies have shown that the inclusion of protease in LP diets can improve growth performance and feed utilization efficiency in weaned piglets [14]. For example, under conditions of reduced dietary protein levels, protease supplementation has been reported to significantly increase ileal amino acid digestibility and improve growth performance in piglets [15,16]. However, not all studies have observed consistent improvements following protease supplementation, suggesting that its efficacy may vary depending on dietary and physiological conditions [17].

Therefore, further evaluation of protease supplementation in LP diets is necessary to better understand its effects on growth performance, nutrient digestibility, and fecal characteristics in weaned piglets. Although protease supplementation has been studied in pig diets, its role in partially compensating for CP reduction under practical LP feeding conditions remains unclear, especially when ATTD and fecal characteristics are not consistently improved. The objective of the present study was to investigate the effects of reducing dietary CP levels and supplementing protease in LP diets on growth performance, nutrient digestibility, and fecal scores in weaned piglets. We hypothesized that protease supplementation in LP diets would partially alleviate the negative effects of reduced dietary CP on growth performance and may influence nutrient digestibility and fecal characteristics in weaned piglets.

## 2. Materials and Methods

### 2.1. Ethics Approval

The animal experiment was reviewed and approved by the Institutional Animal Care and Use Committee of Dankook University, Republic of Korea (Approval No. DK-1-2505_1; approved on 7 February 2025). All procedures involving animals were performed in accordance with the relevant ethical guidelines for animal care and use.

### 2.2. Source of Protease

The protease additive used in the present study was supplied by Morning Bio Co., Ltd. (Cheonan, Republic of Korea) in cooperation with CBS Bio Platforms Inc. (Calgary, AB, Canada). According to the supplier’s information, this additive is a microbial fermentation-derived, multi-component protease preparation developed for use in animal feed. It is intended to support protein degradation across different feed protein sources and to reduce the negative effects of protein-associated anti-nutritional factors. The protease product was thoroughly mixed into the experimental diets at the inclusion rate recommended by the manufacturer.

### 2.3. Study Design

A total of 200 crossbred weaned pigs [Duroc × (Landrace × Yorkshire)] with an initial average BW of 6.01 ± 1.14 kg were selected for this study. The pigs were randomly assigned to four treatment groups using a randomized complete block design based on BW. The feeding trial lasted for 31 days. Each treatment consisted of 10 replicate pens, with five pigs per pen (including two gilts and three barrows). Four dietary treatments were established: CON, basal diet; TRT1, LP diet with CP reduced by 1%; TRT2, LP diet with CP reduced by 2%; and TRT3, TRT2 supplemented with 0.1 g/kg protease. The experiment consisted of three feeding phases: days 1–7, 7–19, and 19–31. Experimental diets were prepared to meet or exceed the nutrient recommendations for weaned pigs established by the National Research Council [18], and their ingredient and nutrient composition are provided in Table 1. Pigs were housed in an environmentally controlled facility fitted with plastic slatted flooring and mechanical ventilation. Each 1.3 m × 1.3 m pen contained a stainless-steel self-feeder and nipple drinkers, providing unrestricted access to feed and water. A 12 h daily lighting schedule was used, and room temperature was maintained at 23 °C. Animals and facility conditions were checked four times per day to verify feed and water availability and to assess pig health.

### 2.4. Growth Performance

During the 31-day experimental period, the BW of each pig was individually measured and recorded at the beginning of the trial and on days 7, 19, and 31 using a portable electronic scale (PB-150, CAS Co., Ltd., Seoul, Korea). Based on these data, ADG was calculated for each treatment group. In addition, daily feed intake and feed refusals were recorded to determine ADFI, which was subsequently used to calculate FCR.

$$FCR=\frac{Feed\;Intake\left(kg\right)}{Weight\;Gain\left(kg\right)}$$

### 2.5. Nutrient Digestibility

Chromium oxide (Cr_2_O_3_) was included in all experimental diets at 0.50% for 7 d before sample collection to determine apparent total tract digestibility (ATTD). Feed samples were placed in sterile bags, while fecal samples were obtained by rectal massage from one gilt and one barrow in each pen at the same time on three consecutive mornings. The fecal material collected during this period was combined by pen. All feed and pooled fecal samples were kept at −20 °C until further analysis.

Before analysis, the samples were dried at 60 °C for 72 h, ground to a fine powder, and passed through a 1 mm screen. DM, N, and E were analyzed according to the procedures of AOAC International [19]. Chromium concentration was quantified using a UV spectrophotometer (Optizen POP, Seoul, Republic of Korea). Energy was measured using a bomb calorimeter (Parr 6100, Moline, IL, USA), and N concentration was determined using a Kjeldahl nitrogen analyzer (Kjeltec 2300, Foss Tecator AB, Hillerød, Denmark). The ATTD was calculated using the following equation:

$$ATTD(\%)=[1-\{(N_{f}\times C_{d})/(N_{d}\times C_{f}\}]\times 100$$

where N_f_ represents nutrient concentration in feces (% DM), C_d_ is chromium concentration in the diet (% DM), N_d_ is nutrient concentration in the diet (% DM), and C_f_ is chromium concentration in feces (% DM).

### 2.6. Fecal Score

On days 7, 19, and 31 of the experiment, fecal scores were determined based on a 5-point scoring system used in previous reports, with the average score calculated from five pigs per pen [20]. The standard of this system is as follows: 1 = hard, dry pellets in a small, hard mass; 2 = hard, formed stool that remains firm and soft; 3 = soft, formed and moist stool that retains its shape; 4 = soft, unformed stool that assumes the shape of the container; 5 = watery, liquid stool that can be poured. Scores were recorded on a pen basis following observations of individual pigs and signs of stool consistency in the pen; all pigs had a mash form of feed.

### 2.7. Statistical Analysis

Statistical analyses were performed using SAS 9.4 software (SAS Institute Inc., Cary, NC, USA). Data normality was evaluated with the Shapiro–Wilk test before further analysis. Variables recorded on days 7, 19, and 31 were assessed separately at each sampling point by one-way analysis of variance. When a significant treatment effect was identified, Tukey’s multiple comparison test was used to compare treatment means. Statistical significance was declared at *p* < 0.05. The pen served as the experimental unit for growth performance, nutrient digestibility, and fecal score data. Data from all pigs that completed the study were included, and no observations were removed from the final analysis.

## 3. Results

### 3.1. Growth Performance

The effects of dietary treatments on growth performance are presented in Table 2. No significant differences in initial BW were observed among treatments (*p* > 0.05). However, compared with the control group, piglets fed the TRT2 showed significantly lower BW on days 7, 19, and 31 (*p* < 0.05).

Similarly, ADG during each phase and over the entire experimental period was significantly reduced in TRT2 (*p* < 0.05). However, protease supplementation partially alleviated the negative effects of reduced CP on growth performance. No significant differences were observed in ADFI or FCR among treatments throughout the experimental period (*p* > 0.05).

### 3.2. Nutrient Digestibility

The effects of dietary treatments on apparent nutrient digestibility are shown in Table 3. The apparent digestibility of DM, N, and E measured on days 7, 19, and 31 was not significantly affected by dietary treatments (*p* > 0.05).

### 3.3. Fecal Scores

The effects of dietary treatments on fecal scores are presented in Table 4. No significant differences in fecal scores were observed among treatments on days 7, 19, and 31 (*p* > 0.05).

## 4. Discussion

During the weaning stage, the digestive system of piglets is not yet fully developed, and the negative effects of reducing dietary CP levels on growth performance have been widely reported [21]. In the present study, reducing dietary CP by 2% resulted in a decrease in BW and ADG, whereas protease supplementation in the LP diet partially restored growth performance. Similar results have been reported in previous studies. For example, it has been demonstrated that supplementation of protease in LP diets significantly increased BW and ADG in weaned piglets, particularly during the early post-weaning phase [22]. In addition, the study by Kim et al. (2021) showed that dietary protease supplementation in LP diets significantly improved ADG and feed efficiency [23]. Therefore, the results of the present study are generally consistent with previous findings, indicating that protease has the potential to improve growth performance under LP dietary conditions.

Regarding nutrient digestibility, no significant differences were observed in the apparent digestibility of DM, N, or E in the present study. Although a partial recovery in growth performance was observed, it was not accompanied by a corresponding improvement in nutrient digestibility. This apparent discrepancy may be explained by the fact that ATTD reflects overall nutrient disappearance at the total-tract level and may not be sensitive enough to detect subtle changes in protein hydrolysis, amino acid release, or nutrient utilization in the small intestine [24]. In addition, growth performance represents the cumulative response of nutrient intake, digestion, absorption, and metabolism over the whole experimental period, whereas ATTD was measured based on fecal samples collected during a specific period [25]. Therefore, improvements in growth performance and changes in ATTD may not always occur in parallel [26]. This finding is consistent with the results of Munezero and Kim (2022), who reported that protease supplementation had limited effects on the overall apparent nutrient digestibility [16]. However, in contrast to the present findings, other studies have reported that protease supplementation can significantly improve CP and amino acid digestibility [27]. For instance, the inclusion of acid protease has been shown to enhance the digestibility of CP and amino acids in soybean meal in piglets [28]. Moreover, another study demonstrated that appropriate levels of protease significantly increased the apparent total tract digestibility of CP and the ileal digestibility of amino acids [29]. These discrepancies may indicate that the efficacy of protease on nutrient digestibility is highly dependent on dietary and physiological conditions. Differences in dietary protein sources, amino acid balance, protease characteristics, inclusion level, substrate specificity, and piglet maturity may contribute to inconsistent responses among studies. Protease efficacy may also depend on the availability of suitable protein substrates and the digestive capacity of piglets [30]. When the basal diet is already highly digestible, the potential for protease to further improve overall nutrient digestibility may be limited [31]. Therefore, the partial recovery of growth performance may not be explained by changes in the ATTD of DM, N, or E. It may instead involve subtle changes in small-intestinal protein degradation, amino acid release and absorption, or nutrient partitioning [32]. Such changes may not be adequately reflected by ATTD measurements. However, because ileal amino acid digestibility, N retention, blood amino acid concentrations, and protein metabolism-related indicators were not measured, these mechanisms remain speculative.

Regarding fecal characteristics, no significant differences were observed among treatments in the present study. Previous studies have also reported that dietary protease supplementation did not significantly affect fecal scores [33], which is consistent with our findings.

Protease supplementation may improve the intestinal environment by enhancing protein digestion and reducing the flow of undigested protein to the hindgut [34]. However, fecal scores primarily reflect fecal consistency and diarrhea status and may not detect subtle changes in protein fermentation, microbial composition, or intestinal barrier function [35]. Moreover, the absence of obvious diarrhea or pathogen challenge in the present study may have limited the detectable response in fecal scores [36]. In contrast, An et al. (2025) reported that protease supplementation reduced the incidence of diarrhea and improved fecal scores as well as intestinal health [37]. These inconsistent findings may be related to differences in experimental duration, health status, and dietary composition. Therefore, although protease supplementation partially restored growth performance, this effect was not reflected in fecal characteristics. Previous studies have suggested that protease may exert its effects by modulating intestinal barrier function and altering the composition of the gut microbiota [38,39], which may partly explain the improvement in growth performance observed in the present study. Because intestinal morphology, barrier function, inflammatory status, and gut microbiota were not evaluated, the potential intestinal effects of protease could not be confirmed. Further studies incorporating these indicators are needed to clarify the mechanisms underlying the effects of protease supplementation.

## 5. Conclusions

Overall, a 2% reduction in dietary CP adversely affected growth performance in weaned piglets, whereas supplementation with 0.1 g/kg protease partially restored BW and ADG. However, because nutrient digestibility and fecal score were not significantly affected, the mechanisms underlying this improvement remain unclear. Therefore, protease supplementation may be a potential nutritional strategy to support growth performance in LP diets, although further studies are needed to clarify its mode of action.

## Figures and Tables

**Table 1:** Composition of weaned piglet diets.

Item	Phase 1			Phase 2			Phase 3		
	PC	NC (CP −1%)	NC (CP −2%)	PC	NC (CP −1%)	NC (CP −2%)	PC	NC (CP −1%)	NC (CP −2%)
Ingredients (%)									
Corn	41.59	44.12	46.55	53.75	56.27	58.77	65.93	68.45	70.91
Soybean meal	18.70	16.17	13.65	18.05	15.54	13.00	18.40	15.87	13.35
Fermented soybean meal	10.00	10.00	10.00	8.00	8.00	8.00	5.00	5.00	5.00
Whey protein	10.00	10.00	10.00	6.00	6.00	6.00	2.00	2.00	2.00
Soy oil	1.68	1.65	1.66	1.84	1.81	1.80	0.98	0.95	0.94
Lactose	13.38	13.38	13.38	8.03	8.03	8.03	3.73	3.73	3.73
Monocalcium Phosphate	1.45	1.48	1.57	1.31	1.32	1.37	1.14	1.17	1.26
Limestone	0.89	0.89	0.88	0.91	0.92	0.92	0.83	0.84	0.82
Lysine (78%)	0.81	0.81	0.81	0.71	0.71	0.71	0.66	0.66	0.66
Methionine (98%)	0.20	0.20	0.20	0.16	0.16	0.16	0.12	0.12	0.12
Threonine (98%)	0.31	0.31	0.31	0.26	0.26	0.26	0.23	0.23	0.23
Tryptophan (98%)	0.04	0.04	0.04	0.03	0.03	0.03	0.03	0.03	0.03
Salt	0.20	0.20	0.20	0.20	0.20	0.20	0.20	0.20	0.20
Mineral mix ^1^	0.20	0.20	0.20	0.20	0.20	0.20	0.20	0.20	0.20
Vitamin mix ^2^	0.20	0.20	0.20	0.20	0.20	0.20	0.20	0.20	0.20
ZnO	0.25	0.25	0.25	0.25	0.25	0.25	0.25	0.25	0.25
Choline (25%)	0.10	0.10	0.10	0.10	0.10	0.10	0.10	0.10	0.10
Total	100.00	100.00	100.00	100.00	100.00	100.00	100.00	100.00	100.00
Calculated value									
Crude protein, %	20.00	19.00	18.00	19.00	18.00	17.00	18.00	17.00	16.00
Metabolizable Energy, kcal/kg	3400	3400	3400	3400	3400	3400	3350	3350	3350
Calcium, %	0.85	0.85	0.85	0.80	0.80	0.80	0.70	0.70	0.70
Phosphorus, %	0.70	0.70	0.70	0.65	0.65	0.65	0.60	0.60	0.60
Lysine, %	1.70	1.63	1.56	1.53	1.46	1.40	1.40	1.34	1.27
Methionine, %	0.49	0.48	0.46	0.44	0.43	0.42	0.40	0.39	0.38
Threonine, %	1.05	1.01	0.97	0.95	0.91	0.87	0.87	0.83	0.79
Tryptophan, %	0.28	0.26	0.25	0.25	0.23	0.22	0.23	0.21	0.20
Crude Fat, %	3.72	3.74	3.79	4.21	4.23	4.27	3.68	3.70	3.74
Crude Fiber, %	1.90	1.86	1.81	2.05	2.00	1.95	2.20	2.15	2.10
Crude Ash, %	5.63	5.53	5.46	5.17	5.07	4.98	4.59	4.50	4.43
Lactose, %	20.00	20.00	20.00	12.00	12.00	12.00	5.00	5.00	5.00

^1^ Provided per kg diet: Fe, 100 mg as ferrous sulfate; Cu, 17 mg as copper sulfate; Mn, 17 mg as manganese oxide; I, 0.5 mg as potassium iodide; and Se, 0.3 mg as sodium selenite. ^2^ Provided per kilograms of diet: vitamin A, 10,800 IU; vitamin D_3_, 4000 IU; vitamin E, 40 IU; vitamin K_3_, 4 mg; vitamin B_1_, 6 mg; vitamin B_2_, 12 mg; vitamin B_6_, 6 mg; vitamin B_12_, 0.05 mg; biotin, 0.2 mg; folic acid, 2 mg; niacin, 50 mg; D-calcium pantothenate, 25 mg.

**Table 2:** Effects of protease supplementation in LP diets on growth performance of weaned piglets ^1^.

Items	CON	TRT1	TRT2	TRT3	SEM ^2^	*p*-Value
Body weight, kg						
Initial	6.01	6.01	6.01	6.01	0.003	0.847
Day 7	7.53 ^a^	7.45 ^ab^	7.32 ^b^	7.43 ^ab^	0.04	0.022
Day 19	12.50 ^a^	12.24 ^ab^	11.77 ^b^	12.17 ^ab^	0.16	0.035
Day 31	18.69 ^a^	18.09 ^ab^	17.28 ^b^	18.09 ^ab^	0.33	0.045
Initial–Day 7						
ADG, g	217 ^a^	206 ^ab^	188 ^b^	203 ^ab^	6	0.022
ADFI, g	261	249	231	252	10	0.210
FCR	1.200	1.203	1.231	1.235	0.012	0.085
Day 7–Day 19						
ADG, g	414 ^a^	399 ^ab^	371 ^b^	393 ^ab^	10	0.048
ADFI, g	557	540	518	549	14	0.261
FCR	1.347	1.355	1.397	1.400	0.018	0.085
Day 19–Day 31						
ADG, g	516 ^a^	488 ^ab^	458 ^b^	493 ^ab^	15	0.048
ADFI, g	817	781	762	799	19	0.214
FCR	1.591	1.605	1.668	1.622	0.030	0.317
Overall						
ADG, g	409 ^a^	390 ^ab^	363 ^b^	389 ^ab^	11	0.045
ADFI, g	591	567	548	579	13	0.158
FCR	1.448	1.458	1.509	1.489	0.018	0.080

^1^ Abbreviation: LP, low-protein; CON: basal diet; TRT1: CP −1%; TRT2: CP −2%; TRT3: TRT2 + 0.1 g/kg protease. ^2^ Standard error of means. ^a,b^ Means in the same row with different superscripts differ (*p* < 0.05).

**Table 3:** Effects of protease supplementation in LP diets on nutrient digestibility in weaned piglets ^1^.

Items, %	CON	TRT1	TRT2	TRT3	SEM ^2^	*p*-Value
Day 7						
Dry matter	88.18	87.22	86.58	87.05	0.52	0.215
Nitrogen	80.19	79.49	78.75	79.02	0.47	0.189
Energy	85.08	84.37	83.25	84.04	0.57	0.196
Day 19						
Dry matter	86.51	85.82	85.06	85.44	0.46	0.194
Nitrogen	80.11	79.70	78.47	79.23	0.57	0.248
Energy	83.10	82.79	81.97	82.40	0.35	0.161
Day 31						
Dry matter	83.02	82.53	82.01	82.34	0.42	0.413
Nitrogen	77.16	76.67	75.78	76.04	0.44	0.160
Energy	79.19	78.58	77.81	78.13	0.45	0.197

^1^ Abbreviation: LP, low-protein; CP, crude protein; CON: basal diet; TRT1: CP −1%; TRT2: CP −2%; TRT3: TRT2 + 0.1 g/kg protease. ^2^ Standard error of means.

**Table 4:** Effects of protease supplementation in LP diets on fecal score in weaned piglets ^1^.

Items	CON	TRT1	TRT2	TRT3	SEM ^2^	*p*-Value
Fecal score						
Day 7	3.58	3.61	3.62	3.60	0.05	0.908
Day 19	3.51	3.55	3.54	3.54	0.04	0.933
Day 31	3.43	3.46	3.46	3.47	0.03	0.814

^1^ Abbreviation: LP, low-protein; CP, crude protein; CON: basal diet; TRT1: CP −1%; TRT2: CP −2%; TRT3: TRT2 + 0.1 g/kg protease. ^2^ Standard error of means.

## Data Availability

For privacy reasons, the data presented in this study are available on request from the corresponding author.

## Abbreviations

The following abbreviations are used in this manuscript:

LP	Low-Protein
N	Nitrogen
CP	Crude Protein
DM	Dry Matter
E	Energy
FCR	Feed Conversion Ratio
ADG	Average Daily Gain
ADFI	Average Daily Feed Intake
AOAC	Association Of Official Analytical Chemists
ATTD	Apparent Total Tract Digestibility
BW	Body Weight
